# Can late gadolinium enhancement on cardiovascular magnetic resonance describe cardiac involvement in patients with systemic sarcoidosis and/or suspect of cardiac involvement of sarcoidosis with cardiac symptoms?

**DOI:** 10.1186/1532-429X-13-S1-P283

**Published:** 2011-02-02

**Authors:** Yufuko Kasai, Nakajima Takatomo, Nomura Arata, Ohmori Hisako, Kimura Fumiko, Sakai Shuji, Hagiwara Nobuhisa

**Affiliations:** 1 Tokyo Women's Medical University, Tokyo, Japan; 2 Saitama Medical University, International Medical Center, Saitama, Japan

## Background

Cardiac involvement of sarcoidosis (CIS) is clinically evident in 5% of patients with systemic sarcoidosis, but CIS are reported to be found at autopsy in 30% to 50%. Some of CIS represents remission and recurrence in clinical setting, and it is difficult to diagnose CIS in cured condition.

## Purpose

We assess whether LGE on CMR reflect CIS in patients with extracardiac sarcoidosis or suspect of CIS due to cardiac symptoms.

## Methods

Forty-three patients (male/female = 12/31, age 30-78 years) with rule out or suspect of CIS with extracardiac sarcoidosis and suspect of CIS without extracardiac sarcoidosis underwent CMR examination. We diagnosed CIS using Diagnostic Standard and Guideline for Sarcoidosis 2006 of the Japanese Society of Sarcoidosis and Other Granulomatous Disorders (2006 criteria) as a gold standard, and subjects were divided into CIS which including suspect of CIS (s/o CIS) and without CIS. We defined CIS as fully satisfied of 2006 criteria, and s/o CIS as follows; satisfied with 1 major and 1 minor of 2006 criteria of CIS with systemic sarcoidosis findings and satisfied with clinical diagnostic criteria of CIS but lack of systemic sarcoidosis findings.

We performed visual segment analysis using standard AHA 17-segments model. The LGE were assessed in each segment, localization (5 patterns: endocardial, mid-wall, epicardial, transmural, and multiple), distribution (3 types: patchy, linear as within 50% of myocardial thickness and band-like as greater than 50% of myocardial thickness), and signal intensity (2 types; lightly, definitively).

## Results

In all 43 patients, 28 patients (65%) showed LGE on CMR. According to the 2006 criteria, 31 patients (72%) divided into CIS including 7 patients of s/o CIS. In CIS, 26 patients (84%) had LGE, and 5 patients (16%) were luck of LGE. The sensitivity, specificity, positive predictive value and negative predictive value of the LGE were 84%, 83%, 92% and 67%, respectively.

Segment analysis of the LGE (n = 442 seg., 26 patients), in localization; epicardial (92 seg., 21patients), transmural (59 seg., 15 patients), endocardial (20 seg., 9 patients), mid-wall (15 seg., 6 patients), and multiple (5 seg., 4 patients) were observed, respectively. In distribution; linear (90 seg., 23 patients), band-like (92 seg., 17 patients) and patchy (4 seg., 4 patients), respectively. And signal intensity, definitively (186 seg. 21 patients) and lightly (18 seg. 8 patients).

## Conclusion

LGE on CMR is a useful diagnostic findings to determine cardiac involvement of sarcoidosis.

